# Governance pathways for scaling neonatal risk stratification tools in LMIC health systems: a multi-site qualitative study in Kenya

**DOI:** 10.3389/fdgth.2026.1883524

**Published:** 2026-07-27

**Authors:** Ronald Danny Nyatuka, Faith Siva, Md Shafiqur Rahman Jabin

**Affiliations:** 1 School of Computing and Engineering Sciences, Strathmore University, Nairobi, Kenya; 2 Department of Medicine and Optometry, Linnaeus University, Kalmar, Sweden

**Keywords:** clinical workflow, digital transformation, health policy, implementation science, institutionalization, organizational readiness

## Abstract

**Background:**

Neonatal mortality remains disproportionately high in low- and middle-income countries (LMICs), where health systems frequently face workforce, infrastructure, and governance constraints. Although neonatal risk stratification tools have demonstrated potential to support the early identification of high-risk newborns, many digital health innovations fail to transition from pilot implementation to sustained institutional practice.

**Objective:**

This study examined governance and health system determinants influencing the scalability of a neonatal risk stratification tool across Kenyan health facilities and identified policy-relevant pathways for sustainable scale-up in resource-constrained settings.

**Methods:**

A multi-site qualitative implementation study was conducted across three Kenyan health facilities implementing a paper-based neonatal risk stratification tool during pilot deployment. Semi-structured key informant interviews, field observations, and implementation reflections were used to examine governance alignment, workflow integration, infrastructure readiness, staffing capacity, financing considerations, and sustainability challenges. Data were analyzed using reflexive thematic analysis informed by implementation science frameworks, including the Consolidated Framework for Implementation Research (CFIR) and the Nonadoption, Abandonment, Scale-up, Spread, and Sustainability (NASSS) framework.

**Results:**

Implementation sustainability was shaped primarily by system-level determinants rather than technological functionality alone. Three major themes emerged: (1) governance fragmentation constrained institutional integration through parallel documentation systems and administrative approval pathways; (2) structural capacity limitations, including workforce shortages, infrastructure instability, and financing uncertainty, reduced implementation sustainability; and (3) adaptive hybrid implementation approaches facilitated transitional scale-up within resource-constrained environments. Leadership engagement, contextual adaptation, and phased implementation strategies emerged as important enabling factors influencing implementation feasibility and organizational integration.

**Conclusions:**

Scaling neonatal risk stratification tools in LMIC health systems requires governance-oriented, system-sensitive implementation strategies that extend beyond technology deployment alone. Sustainable institutionalization depends on alignment across workforce capacity, infrastructure readiness, financing mechanisms, workflow integration, and policy coordination. Hybrid and phased implementation approaches may offer pragmatic pathways to scale neonatal digital health innovations in resource-constrained settings.

## Introduction

### Neonatal mortality and the need for early risk identification

Neonatal mortality remains a major contributor to under-five mortality globally, with the highest burden concentrated in low- and middle-income countries (LMICs) (1–3). Despite improvements in maternal and newborn care, many neonatal deaths remain preventable through earlier identification of clinical deterioration and timely intervention (4). In resource-constrained neonatal settings, delayed recognition of high-risk neonates is frequently compounded by staffing shortages, inconsistent documentation practices, limited monitoring capacity, and fragmented clinical workflows.

### Emergence of neonatal risk stratification tools

Machine learning (ML)-driven neonatal risk stratification tools have increasingly been explored to support early risk identification and clinical decision-making (5, 6). These tools have the potential to strengthen the prioritization of care, improve recognition of vulnerable neonates, and support more structured clinical assessment in high-burden environments. However, while many predictive models demonstrate acceptable technical performance during pilot implementation, translation into routine clinical practice remains challenging (7, 8).

### Governance and institutionalization challenges in LMIC health systems

The implementation gap between pilot innovation and sustainable institutional adoption is particularly pronounced in LMIC health systems, where governance complexity, workforce shortages, infrastructure instability, and financing constraints may impede long-term integration even when frontline usability is favorable (9, 10). Previous digital health research has shown that technologies insufficiently aligned with organizational workflows, documentation systems, and institutional governance structures may introduce operational strain rather than implementation efficiency (11–14).

Within neonatal care settings, these challenges may be amplified by high patient acuity, rapid clinical decision-making, and substantial documentation demands. Introducing additional clinical tools into such environments, therefore, requires alignment not only with frontline workflows but also with broader health system structures, including leadership support, financing pathways, policy integration, and implementation governance.

### Implementation science and health system scale-Up

Implementation science frameworks increasingly emphasize the importance of organizational and contextual determinants in shaping implementation outcomes. The Consolidated Framework for Implementation Research (CFIR) identifies leadership engagement, organizational culture, resource availability, and policy environment as major determinants influencing implementation success (15). Similarly, the Nonadoption, Abandonment, Scale-up, Spread, and Sustainability (NASSS) framework highlights the sociotechnical complexity that influences the long-term sustainability of digital health innovations (16).

Despite a growing implementation science literature, relatively little attention has been paid to governance-oriented pathways that influence how neonatal digital health innovations transition from pilot implementation to sustainable institutional scale-up in LMIC health systems. Much of the existing literature remains concentrated on technical feasibility or user-level adoption rather than broader institutionalization processes.

Although previous research has demonstrated that governance, workforce capacity, infrastructure readiness, and organizational context influence the implementation of digital health innovations, these determinants have largely been examined as individual barriers or facilitators of technology adoption and usability (16–18). Comparatively little attention has been given to how these system-level factors interact during the transition from successful pilot implementation to sustainable institutionalization, particularly in neonatal care within resource-constrained LMIC health systems (19). Addressing this gap, the present study integrates empirical findings with the CFIR and NASSS frameworks to examine how governance coordinates leadership engagement, workflow integration, documentation practices, workforce capacity, and organizational readiness during scale-up (17, 20). By adopting this governance-oriented perspective, the study provides a more comprehensive understanding of the mechanisms that shape the long-term institutionalization of neonatal digital health innovations and offers practical insights to inform implementation strategies in comparable health systems.

### Study Aim

Building on prior implementation work evaluating the operational feasibility and frontline usability of a neonatal risk stratification tool in Kenyan neonatal settings, this study shifts the analytical focus to governance and system-level determinants that influence scalability. The aim of this study is to examine governance and health system factors that affect the scale-up of neonatal risk stratification tools across Kenyan health facilities and to identify policy-relevant pathways to support sustainable implementation in resource-constrained health systems.

## Methods

### Study design

This study employed a multi-site qualitative implementation design to examine governance and health system determinants influencing the scalability of a neonatal risk stratification tool across Kenyan health facilities. The study was part of a broader neonatal implementation project evaluating the operational feasibility, usability, and system-level integration of a paper-based neonatal risk stratification tool intended to support the early identification of high-risk neonates during the first 48 h of care. Unlike prior usability-focused analyses within the project, the present study specifically examined organizational, governance, and structural determinants that influence sustainability and scale-up readiness.

The study design was informed by implementation science approaches that emphasize contextual and organizational determinants of implementation outcomes (15, 17, 21). In addition, the study drew conceptually from the Nonadoption, Abandonment, Scale-up, Spread, and Sustainability (NASSS) framework to support the interpretation of sustainability and institutionalization processes associated with digital health innovations (16).

### Study setting

The study was conducted at three Kenyan health facilities that were implementing the neonatal risk stratification tool during its pilot deployment. The facilities represented diverse neonatal care environments with variation in staffing capacity, infrastructure stability, governance structures, documentation systems, and ICT maturity. All participating facilities provided inpatient neonatal services within resource-constrained settings and managed varying levels of neonatal acuity and patient volume.

The neonatal risk stratification tool evaluated in this study was implemented as a structured, paper-based clinical support and documentation tool that incorporates routinely collected neonatal predictors to support early identification of high-risk neonates. The pilot implementation intentionally used a paper-based format to accommodate differences in digital infrastructure readiness across facilities.

### Participants and sampling

Purposive sampling was used to recruit participants directly involved in implementation oversight, operational coordination, or clinical use of the neonatal risk stratification tool. One purposively selected key informant was recruited from each participating facility to represent a distinct organizational perspective relevant to implementation. The participants comprised a neonatal unit leadership representative responsible for clinical oversight and workflow coordination, an administrative and implementation representative responsible for governance and operational coordination, and a health information and clinical implementation representative responsible for documentation and implementation support. All participants were directly involved in implementation oversight and organizational decision-making related to the neonatal risk stratification tool.

Purposive sampling was guided by the principle of information power, which suggests that a smaller sample may be appropriate when the study aim is focused, participants possess highly specific expertise, and the analysis is supported by established theoretical frameworks (22). Each participant held a distinct leadership or implementation role at one of the participating facilities and was selected for their direct involvement in implementation oversight, organizational decision-making, or operational coordination of the neonatal risk stratification tool. Recruitment concluded when the available participants with these predefined roles had been interviewed, and the collected data were considered sufficient to address the study objectives through rich, context-specific information rather than numerical saturation. Recruitment continued until sufficient depth and variation of implementation perspectives were achieved across sites and professional roles. The characteristics of the study participants are summarized in Table 1.

**Table 1:** Participant characteristics.

Participant Role	Facility Type	Primary Implementation Role
Neonatal unit leadership representative	Facility 1	Clinical oversight and workflow coordination
Administrative/implementation representative	Facility 2	Governance and operational coordination
Health information/clinical implementation representative	Facility 3	Documentation and implementation support

### Data collection

Data were collected during the pilot implementation phase using semi-structured key informant interviews, field observations, and implementation reflections documented during deployment. Interview guides explored leadership engagement, workflow integration, staffing constraints, governance alignment, financing considerations, infrastructure readiness, documentation systems, and sustainability challenges associated with implementation and scale-up.

Interviews were conducted in person using a semi-structured interview guide and lasted approximately 40–60 min. With participant consent, interviews were audio-recorded, transcribed verbatim, and anonymized prior to analysis. Field observations were additionally conducted during routine neonatal care activities to contextualize workflow dynamics, staffing pressures, documentation practices, and operational constraints across facilities.

Researcher reflexivity was maintained throughout data collection and analysis through documentation of contextual observations, implementation experiences, and analytic reflections. These reflexive notes were used to support critical examination of emerging interpretations and to enhance transparency during theme development by encouraging ongoing reflection on how researcher perspectives might influence data interpretation.

The lead researcher maintained reflexive awareness throughout the study process by documenting implementation observations and contextual reflections during data collection and analysis. Triangulation of interview data, field observations, and implementation reflections strengthened interpretive credibility and contextual understanding of implementation processes.

### Data analysis

Data were analyzed using reflexive thematic analysis following Braun and Clarke (2021). Analysis was informed by the Consolidated Framework for Implementation Research (CFIR), which emphasizes the importance of organizational and contextual determinants influencing implementation success (15).

Analysis followed an iterative process in which interview transcripts, field observations, and implementation reflections were repeatedly reviewed to build familiarity with the data. Initial inductive codes were developed from the raw data, then iteratively grouped into candidate categories and themes. Throughout the analytical process, themes were continuously refined through comparison across the three participating facilities and interpreted in relation to the CFIR and NASSS frameworks. Regular discussions among the research team were undertaken to challenge interpretations, enhance analytical consistency, and ensure that the final themes accurately reflected the empirical data.

The analytical process proceeded through iterative familiarization with transcripts and field notes, open coding, development of candidate themes, thematic refinement, and cross-case comparison across facilities. Initial coding was conducted inductively to identify emerging structural and governance-related determinants from the data. Subsequently, themes were interpreted in relation to implementation science constructs, particularly CFIR domains relating to inner-setting and outer-setting determinants.

Cross-case synthesis was used to compare implementation experiences and readiness profiles across facilities and to identify both shared and context-specific determinants influencing scalability and institutionalization (23). Emerging interpretations were discussed among the research team to strengthen interpretive consistency and analytical credibility.

In addition to thematic interpretation, the final themes were synthesized into conceptual visual models to illustrate the relationships among the implementation determinants identified across the participating facilities. These figures were developed iteratively by the research team following completion of the thematic analysis and were informed by the CFIR and NASSS frameworks. The visual models were not analyzed as independent data; rather, they were constructed to summarize and communicate the conceptual relationships emerging from the empirical findings.

### Ethical considerations

Ethical approval (SU-ISERC2949/25) was obtained from the Strathmore University Institutional Scientific Ethics Review Committee (SU-ISERC), Nairobi, Kenya, on 28 July 2025, prior to commencement of the study. The study also complied with relevant national ethical and research governance requirements for health research in Kenya.

All participants provided informed consent before participation after receiving information regarding the study aims, voluntary participation, confidentiality protections, and data management procedures. Interview transcripts were anonymized before analysis to protect participant confidentiality and institutional privacy. The study adhered to established ethical principles for qualitative health systems research throughout data collection, analysis, and reporting.

## Results

### Participant characteristics

A total of three key informant interviews (KIIs) were conducted across the participating facilities. Participants represented leadership and implementation-related roles directly involved in neonatal care delivery, implementation coordination, and operational decision-making during pilot deployment of the neonatal risk stratification tool.

The themes presented below represent the perspectives of one purposively selected key informant from each participating facility. Although each participant occupied a different leadership or implementation role, the analysis focused on identifying shared and context-specific organizational perspectives across the three facilities rather than quantifying individual opinions.

Participants provided perspectives on governance alignment, staffing constraints, infrastructure readiness, workflow integration, implementation coordination, and sustainability considerations associated with scaling up the neonatal risk stratification tool.

### Governance fragmentation and institutional integration challenges

Across the three facilities, informants described governance-related barriers that influence the long-term institutionalization of the neonatal risk stratification tool. Across facilities, implementation feasibility was affected by documentation governance structures, approval pathways, and the coexistence of parallel reporting systems. The three informants reported that introducing additional documentation tools required administrative negotiation and alignment with existing institutional reporting requirements.

Informants from multiple facilities noted that the presence of parallel documentation systems contributed to implementation complexity and reduced the feasibility of sustained routine integration. In some facilities, staff perceived duplication of documentation tasks as a barrier to long-term adoption, particularly during periods of increased patient volume.

One participant explained:

“If the county approves and says go ahead, then implementation becomes easier for us at the facility level.” (Facility 1)

Leadership endorsement emerged as an important enabling factor influencing implementation momentum and staff engagement. Facilities demonstrating stronger managerial support appeared more willing to accommodate workflow adaptation and to coordinate implementation during the pilot phase.

These findings suggested that implementation sustainability was influenced not only by frontline acceptability but also by broader institutional governance structures and administrative integration processes.

### Structural capacity constraints and sustainability challenges

Participants across all facilities described workforce shortages and fluctuating staffing levels as major barriers influencing implementation sustainability. Documentation tasks associated with the neonatal risk stratification tool frequently competed with urgent clinical responsibilities during periods of high patient acuity and staff shortages.

As one participant noted:

“Ideally one nurse should care for two patients, but currently one nurse may handle up to ten patients.” (Facility 3)

Infrastructure instability additionally influenced implementation feasibility across facilities. Participants described inconsistent access to ICT infrastructure, variable digital readiness, and resource limitations that constrained opportunities for immediate digital transition. While participants generally viewed future digital integration positively, several emphasized that current infrastructure limitations restricted the feasibility of fully digital deployment during the pilot phase.

Financing uncertainty was also identified as a potential barrier to scale-up. Participants highlighted concerns relating to the long-term sustainability of printing, training, supervision, and implementation coordination if external implementation support were withdrawn.

Overall, findings demonstrated that implementation sustainability depended substantially on broader structural capacity rather than technological functionality alone.

### Adaptive hybrid strategies for transitional scale-Up

Despite implementation constraints, participating informants identified several adaptive strategies that supported operational feasibility during the pilot phase. Hybrid implementation approaches that combine paper-based workflows with gradual digital transition planning were viewed as pragmatic in resource-constrained settings where ICT infrastructure remained variable.

One participant commented:

“We are currently using Afya One, Excel, and some paperwork. So it is a combination of digital systems and manual records.” (Facility 3)

Participants additionally emphasized the importance of phased onboarding, contextual adaptation, and continuous implementation feedback during early adoption. Structured orientation and ongoing mentorship were perceived as important for reducing implementation uncertainty and supporting workflow integration among newly rotating staff.

Cross-site comparisons further demonstrated that facilities with stronger leadership engagement and implementation flexibility were better able to adapt workflows to accommodate the neonatal risk stratification tool during pilot deployment.

**Figure 1:**
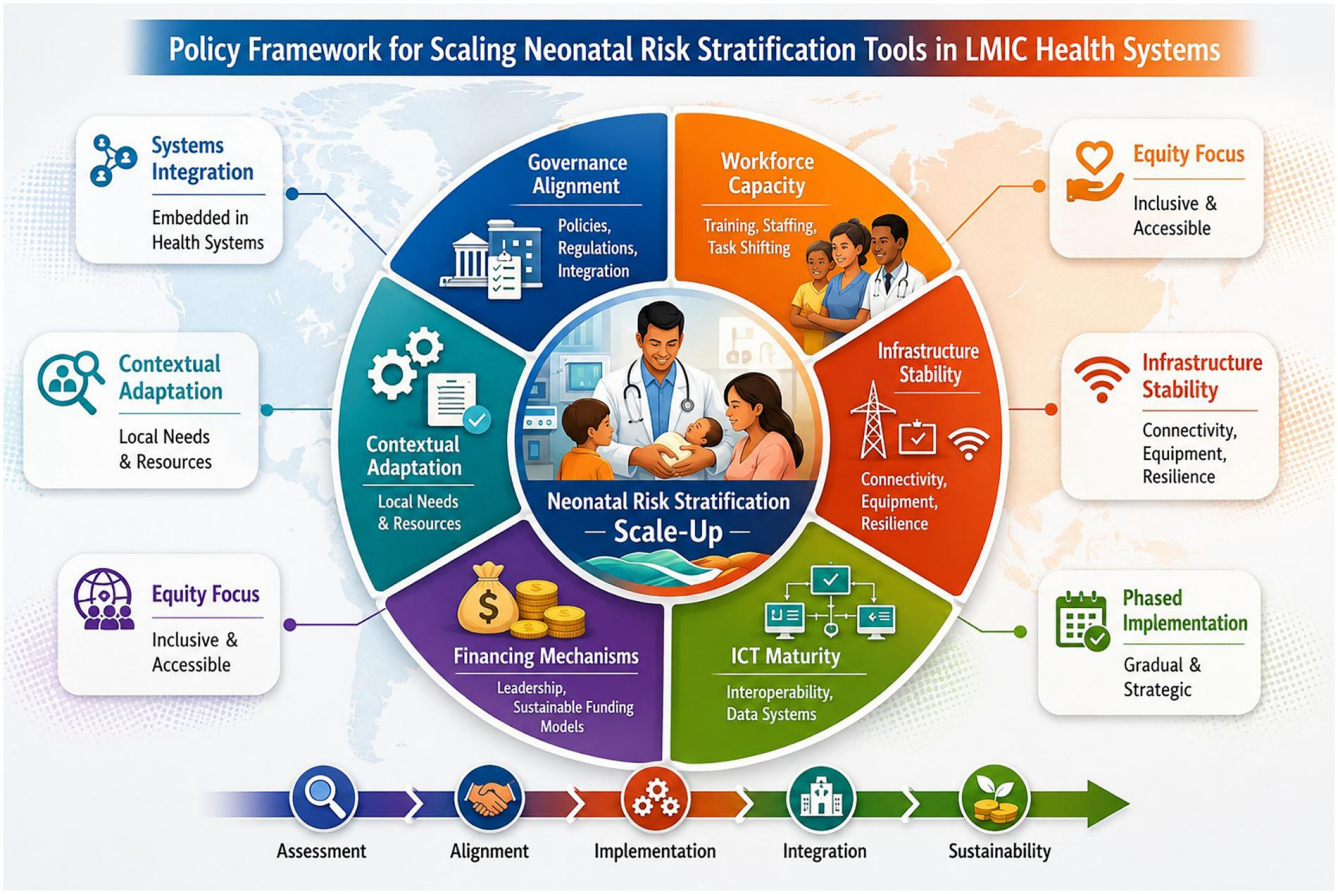
System-Level bottlenecks and enablers for scaling neonatal digital tools in LMIC health systems.

To facilitate interpretation of the qualitative findings, we synthesized the relationships among the identified themes into a conceptual model (Figure 1). Figure 1 summarizes the major structural barriers and enabling conditions influencing implementation sustainability and scale-up across participating facilities.

This conceptual figure was developed by synthesizing qualitative findings and illustrates the principal governance, organizational, and structural determinants identified across the participating facilities. The relationships depicted represent the authors’ interpretation of the empirical findings, informed by the CFIR and NASSS implementation science frameworks.

### Cross-Case synthesis of scale-Up determinants

Cross-case synthesis demonstrated that implementation sustainability was shaped by the interaction between governance alignment, structural capacity, and contextual adaptation. Although frontline acceptance of the neonatal risk stratification tool was generally favorable during the pilot implementation, long-term institutionalization appeared contingent on broader organizational readiness and mechanisms for policy integration.

Facilities with stronger administrative engagement, greater workflow flexibility, and more stable staffing structures demonstrated comparatively greater implementation adaptability. Conversely, facilities experiencing greater staffing instability and operational pressure reported increased challenges integrating additional documentation processes into routine neonatal care workflows.

Collectively, the findings indicate that successful scale-up of neonatal risk stratification tools within LMIC health systems requires governance-oriented implementation approaches that simultaneously address workforce capacity, infrastructure readiness, financing sustainability, and institutional integration, rather than focusing exclusively on technological deployment.

## Discussion

The present study is part of a broader mixed-methods implementation research program evaluating a machine-learning-derived neonatal risk predictor in Kenyan health facilities. The overall project was designed as a phased evaluation, including a published study protocol, an assessment of usability, feasibility, acceptability, and workflow integration, and the present qualitative investigation of governance and health system determinants influencing sustainable scale-up (3). Whereas the earlier studies primarily examined frontline implementation experiences and user-centered evaluation of the neonatal risk predictor, the current study extends this evidence by focusing on the organizational, governance, and system-level mechanisms that influence the transition from successful pilot implementation to long-term institutionalization. Together, these complementary studies provide a comprehensive understanding of both implementation processes and the broader health system conditions required for sustainable scale-up of neonatal digital health innovations in LMICs.

### Principal findings

This study examined the determinants of governance and the health system that influence the scalability of a neonatal risk stratification tool across Kenyan health facilities. The findings demonstrate that long-term implementation sustainability was shaped less by technological functionality alone and more by broader structural and governance conditions influencing institutional integration. Workforce instability, fragmented documentation systems, infrastructure limitations, financing uncertainty, and leadership engagement emerged as major determinants influencing implementation feasibility and scalability.

Beyond confirming previously recognized implementation challenges, this study contributes an integrated governance-oriented interpretation of implementation sustainability in neonatal digital health. Rather than treating governance, workforce capacity, infrastructure readiness, financing, and leadership engagement as separate determinants of implementation, our findings demonstrate that these factors function as interconnected components of organizational readiness, collectively influencing the institutionalization of digital innovations. This perspective extends existing implementation science by illustrating the dynamic relationships among system-level determinants during scale-up and by proposing a governance pathway that can inform implementation planning in comparable resource-constrained health systems.

Successful scale-up depended on the alignment of clinical workflows, governance structures, organizational readiness, and broader health system capacity, reinforcing implementation science evidence that contextual and organizational determinants extend beyond technological performance (17). Rather, successful scale-up appears to depend on alignment among clinical workflows, governance structures, organizational readiness, and broader health system capacity. These findings are consistent with the implementation science literature, which emphasizes that implementation success depends on contextual and organizational determinants that extend beyond technological performance (16, 17, 21).

Importantly, this study extends the existing implementation science literature by demonstrating that governance should not be viewed merely as an external contextual determinant but as an integrating mechanism through which workforce capacity, infrastructure readiness, financing, workflow integration, and leadership engagement collectively influence implementation sustainability (16, 17). Rather than acting independently, these determinants interact dynamically to shape organizational readiness and institutionalization. This governance-oriented perspective offers a more integrated explanation of scale-up than studies that primarily focus on technological performance or user acceptance, providing a practical conceptual lens for planning the sustainable implementation of neonatal digital health innovations in LMIC health systems.

These findings also extend the existing literature by shifting the analytical focus from individual determinants of implementation to the interactions among governance, organizational readiness, and health system capacity during implementation. Whereas previous studies have predominantly evaluated digital health innovations in terms of usability, feasibility, or technology adoption, our findings demonstrate that long-term institutionalization is shaped by coordinated governance processes that influence documentation practices, leadership engagement, workforce capacity, and organizational adaptation simultaneously. This broader systems perspective contributes to implementation science by illustrating how governance serves as an organizing mechanism linking multiple implementation determinants during scale-up in LMIC health systems.

### Governance fragmentation and the pilot-to-scale Gap

A major finding of this study was the impact of governance fragmentation on the sustainability of implementation. Across facilities, parallel documentation systems, administrative approval requirements, and fragmented reporting structures complicated the integration of the neonatal risk stratification tool into routine clinical workflows. These findings align with broader digital health literature demonstrating that technologies insufficiently integrated into institutional governance structures frequently encounter operational resistance during scale-up (10, 24–26).

The findings additionally reinforce growing recognition that pilot implementation success does not necessarily translate into sustainable institutional adoption. Previous implementation studies have described a persistent “pilot-to-scale gap” in digital health, whereby interventions demonstrating feasibility in controlled pilot contexts fail to achieve broader institutional integration (7, 8, 27, 28). In the present study, the neonatal risk stratification tool was generally perceived as clinically valuable; however, its sustainability depended on broader governance alignment, leadership engagement, and institutional coordination mechanisms.

Beyond creating additional administrative requirements, governance fragmentation appeared to influence implementation through several interconnected mechanisms. Fragmented governance structures limited coordination between clinical and administrative processes, resulting in parallel documentation systems, inconsistent implementation expectations, and delayed organizational decision-making. These challenges increased documentation burden, reduced opportunities for workflow integration, and constrained leadership capacity to coordinate implementation activities across organizational levels (17, 29) Consequently, governance fragmentation functioned not simply as an isolated implementation barrier but as a system-level mechanism that amplified the effects of workforce shortages, infrastructure limitations, and financing uncertainty, thereby reducing organizational readiness for sustainable scale-up. This interpretation highlights the interdependence of implementation determinants and reinforces the importance of adopting a systems-oriented approach when planning the institutionalization of digital health innovations (29–31).

These findings further support previous research demonstrating that inadequate governance alignment and fragmented implementation processes may contribute to unintended operational strain during digital health implementation (30–34). Collectively, these findings demonstrate that governance influences scale-up by coordinating organizational decision-making, workflow integration, and resource allocation, thereby shaping the conditions necessary for sustainable implementation.

### Workforce capacity and workflow integration

Workforce instability emerged as one of the most significant structural barriers influencing implementation sustainability. Across the three facilities, informants described how staffing shortages and high patient acuity constrained the feasibility of integrating additional documentation processes into routine neonatal workflows. In high-burden neonatal environments, documentation tasks frequently compete directly with urgent clinical responsibilities.

These findings align with previous studies demonstrating that cognitive workload, staffing pressures, and workflow fragmentation substantially influence the adoption of digital health interventions and clinical documentation systems (3, 35–38). Similar observations have also been reported in studies examining electronic health record integration and workflow adaptation within complex clinical environments (39, 40).

The findings additionally reinforce principles from Normalization Process Theory, which emphasizes that implementation sustainability depends on whether innovations can be effectively integrated into routine operational practices (41–45). Without sufficient workforce capacity or workflow adaptation, clinically beneficial innovations may remain operationally fragile despite positive user perceptions.

Importantly, the present findings extend prior usability-focused analyses from this broader neonatal implementation project by demonstrating that workflow integration challenges persist even when frontline acceptance is favorable. This distinction highlights the importance of examining implementation not only at the user level but also at organizational and system levels simultaneously.

### Hybrid implementation as a pragmatic transitional strategy

Infrastructure instability and variability in ICT readiness influenced implementation feasibility across participating facilities. While participants generally supported future digital integration, many emphasized that a fully digital implementation was not yet operationally feasible within existing infrastructure. Hybrid approaches that combine paper-based workflows with phased digital transition planning therefore emerged as pragmatic implementation pathways.

These findings align with previous LMIC digital health research demonstrating that premature digitalization in resource-constrained settings may lead to operational fragility when infrastructure capacity, interoperability, or technical support systems are insufficient (9, 42, 46). Similar concerns regarding operational disruption associated with insufficiently supported digital implementation have also been reported in previous health information technology incident analyses (33, 47, 48).

Rather than viewing hybrid implementation as a temporary compromise, the findings suggest that phased implementation strategies may represent adaptive and contextually appropriate models for scale-up within resource-constrained health systems. This observation is consistent with emerging implementation science literature emphasizing contextual adaptation, iterative implementation, and flexible integration approaches during digital transformation processes (16, 49).

**Figure 2:**
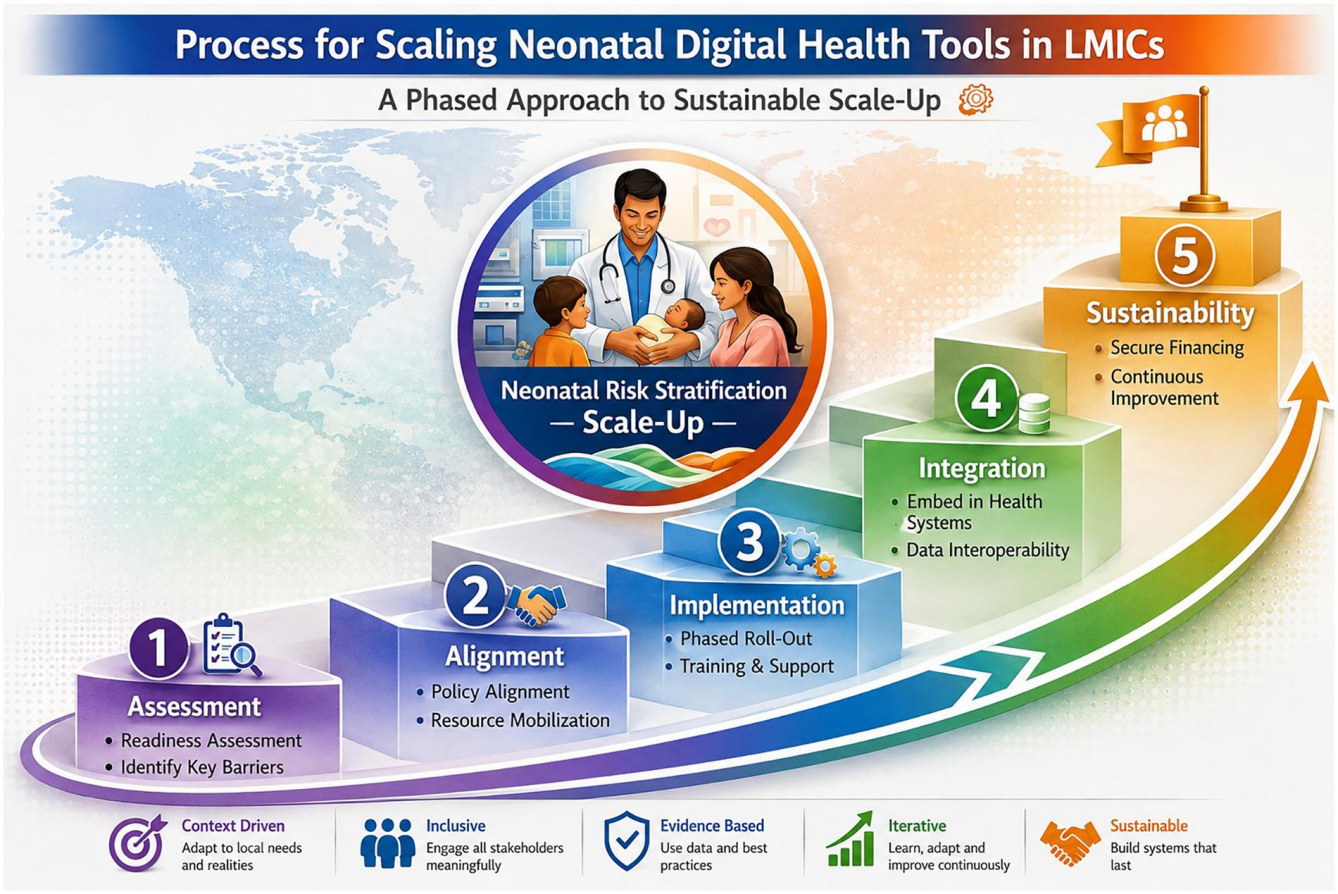
Policy framework for scaling neonatal risk stratification tools in LMIC health systems.

Building on these findings, Figure 2 presents a conceptual governance framework synthesized from the qualitative analysis. Figure 2 presents the governance and policy domains identified as central to the sustainable institutionalization of neonatal risk stratification tools in LMIC health systems.

This conceptual framework was derived from the qualitative themes identified in the study and illustrates the governance and health system domains considered essential for sustainable implementation and institutionalization. The framework synthesizes the empirical findings through the lens of the CFIR and NASSS frameworks.

### Leadership engagement and institutionalization

Leadership engagement emerged as an important enabling factor influencing implementation coordination and organizational adaptability. Facilities demonstrating stronger administrative support appeared better able to accommodate workflow adjustments, coordinate implementation, and engage staff during the pilot deployment.

These findings align with implementation science literature, identifying leadership engagement as a central determinant of implementation climate and organizational readiness (50–52). Leadership support may be particularly important in resource-constrained settings, where implementation often competes with existing operational pressures and limited institutional resources. The findings additionally suggest that leadership engagement contributes not only to implementation authorization but also to institutional normalization processes by signaling strategic value, legitimizing workflow adaptation, and facilitating resource prioritization during implementation phases.

### Implications for scale-Up in LMIC health systems

Collectively, the findings indicate that scaling neonatal risk stratification tools in LMIC health systems requires more than the deployment of a digital innovation (53). Successful institutionalization depends on governance mechanisms that support coordinated decision-making across clinical, administrative, and policy levels. Specifically, the findings highlight the importance of integrating new documentation processes into existing clinical workflows, establishing clear leadership accountability for implementation, adopting phased implementation strategies that reflect local infrastructure readiness, and strengthening workforce capacity through continuous training and organizational support. These governance mechanisms were consistently identified across the participating facilities as important facilitators of sustainable implementation and provide practical priorities for future scale-up initiatives in comparable resource-constrained settings.

These observations support increasing calls within implementation science and digital health literature for stronger integration between technological innovation and health system strengthening approaches (10, 24). From an implementation science perspective, these findings suggest that successful scale-up depends less on addressing individual implementation barriers than on strengthening the relationships among governance, organizational capacity, and clinical workflows. Improvements in one domain are unlikely to achieve sustained implementation unless complementary organizational conditions are also established. This systems perspective extends the interpretation of the findings beyond individual determinants and emphasizes implementation as a dynamic organizational process rather than a series of independent barriers. These findings suggest that implementation strategies focused primarily on technical deployment, without parallel investments in governance structures, leadership engagement, workforce capacity, and workflow integration, are unlikely to achieve sustainable institutionalization in resource-constrained neonatal care settings.

**Figure 3:**
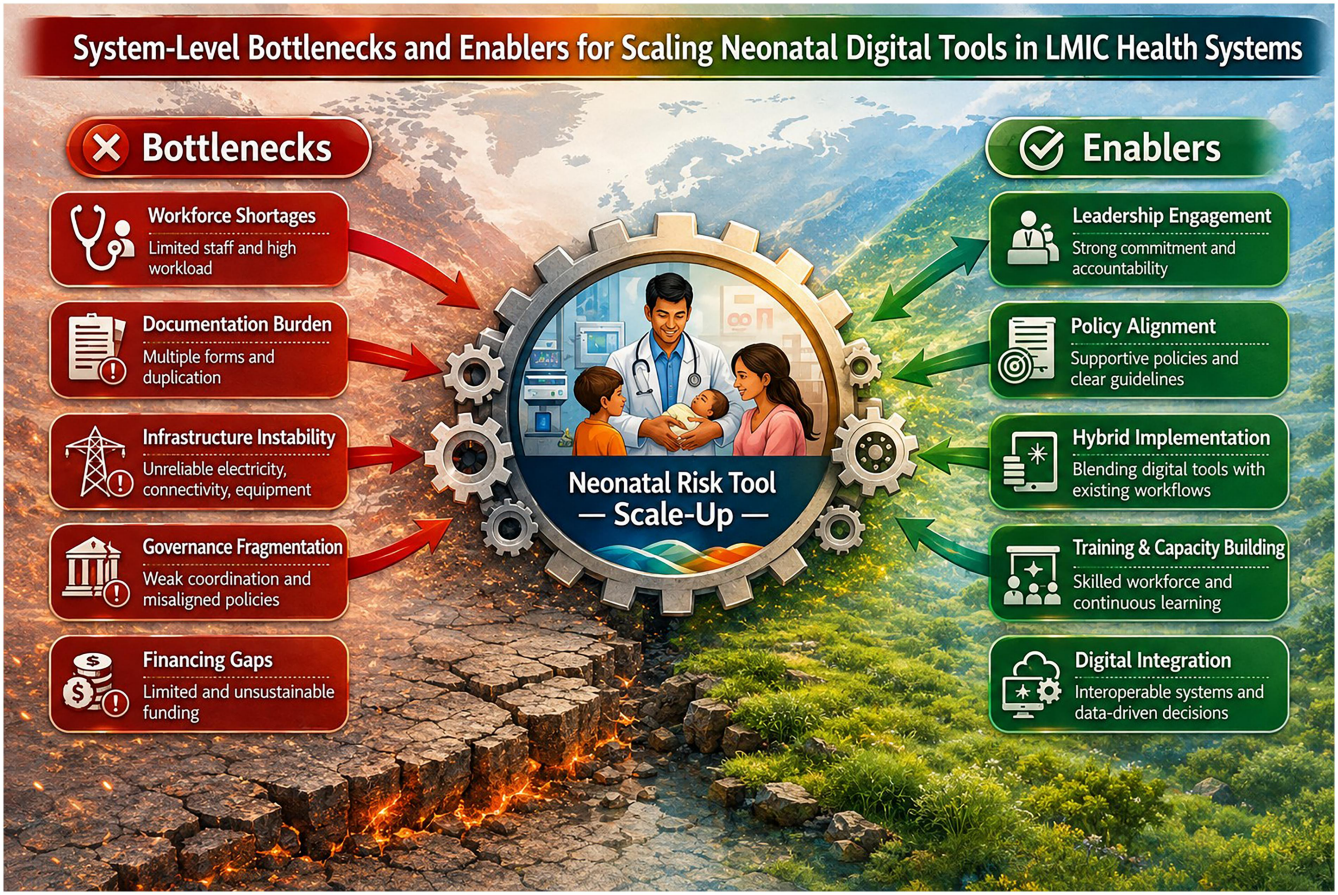
Phased scale-Up pathway for neonatal digital health innovations in LMIC health systems.

Figure 3 integrates the study findings into a proposed phased implementation pathway to support sustainable scale-up in comparable LMIC settings.

This conceptual pathway was developed from the study findings to illustrate a staged implementation process progressing from pilot implementation to sustainable institutionalization. The phases represent an interpretive synthesis of the qualitative data rather than a prescriptive implementation model.

### Strengths and limitations

This study has several important strengths. First, the study examined implementation scalability through a governance and health systems lens rather than focusing exclusively on user-level usability or technological performance. This broader implementation perspective aligns with the growing calls within implementation science and digital health literature to evaluate organizational, contextual, and policy determinants that influence sustainability and institutionalization (10, 16, 21). Second, the multi-site design enabled cross-case comparisons across facilities with differing operational contexts, thereby strengthening contextual interpretation of implementation determinants and enhancing transferability of findings within comparable LMIC neonatal settings (23). Third, triangulation of interviews, field observations, and implementation reflections enhanced analytical depth and interpretive credibility, which is considered an important methodological strength within qualitative implementation research (24, 54).

In addition, the study contributes to the relatively limited body of evidence examining governance and institutionalization challenges associated with neonatal digital health implementation in resource-constrained settings. The findings further extend previous work examining operational and sociotechnical challenges associated with digital health implementation and workflow integration (14, 33, 55).

However, several limitations should also be considered. The small number of leadership-level interviews limited representativeness and restricted the breadth of perspectives captured across the participating facilities. Broader inclusion of county-level policymakers, financial officers, and national digital health stakeholders would have strengthened policy-level analysis and improved understanding of the financing and governance pathways that influence scale-up. In addition, the study examined implementation during the pilot deployment and therefore could not assess long-term sustainability, normalization, or post-scale-implementation outcomes. Longitudinal implementation studies are increasingly recognized as important for understanding the dynamics of sustainability within digital health systems (16, 50).

As with qualitative implementation research more broadly, interpretation may also have been influenced by contextual subjectivity and implementation-stage dynamics (54, 56, 57). Furthermore, variability in infrastructure readiness and staffing conditions across facilities may limit the transferability of findings to settings with substantially different organizational or resource conditions. Accordingly, the governance-level findings should be interpreted as contextually grounded implementation insights generated from information-rich leadership perspectives rather than as statistically generalizable representations of all neonatal care settings.

### Future research directions

Future research should examine long-term institutionalization processes associated with neonatal digital health innovations following broader implementation and policy integration. Larger multi-site implementation studies involving county-level policymakers, digital health coordinators, and national health system stakeholders would strengthen understanding of the governance pathways that influence sustainable scale-up and policy integration within LMIC health systems (16, 21).

Additional research should also evaluate phased hybrid implementation models combining paper-based and digital workflows in resource-constrained settings. Comparative studies examining interoperability, financing sustainability, workforce adaptation, and implementation outcomes across differing implementation modalities may further inform pragmatic digital transition strategies within LMIC neonatal health systems (9, 42).

Further implementation-effectiveness research linking governance readiness, workflow integration, and downstream neonatal outcomes would strengthen the evidence regarding the broader health system impact of neonatal risk stratification tools during scale-up and institutionalization. Hybrid effectiveness-implementation designs and longitudinal implementation evaluations may be particularly valuable for examining how implementation determinants evolve over time and influence sustainability within routine neonatal care environments (58, 59).

Future work should additionally explore cost-effectiveness, workforce implications, and interoperability requirements associated with scaling neonatal digital health innovations across heterogeneous health systems. Such evidence may support policymakers and health system leaders in developing context-sensitive implementation strategies that balance technological innovation with organizational readiness and sustainability considerations (24, 28).

## Conclusion

This study demonstrates that the successful scale-up of neonatal risk stratification tools in resource-constrained LMIC health systems depends on governance mechanisms that align leadership, clinical workflows, documentation systems, workforce capacity, financing, and infrastructure readiness throughout implementation. Rather than addressing these determinants independently, implementation strategies should promote coordinated organizational planning and phased integration that reflects local health system capacity. By identifying specific governance and organizational mechanisms that influence institutionalization, this study provides practical guidance for policymakers, hospital leaders, and implementation teams seeking to support the sustainable adoption of neonatal digital health innovations. Workforce instability, fragmented documentation systems, infrastructure limitations, financing uncertainty, and governance alignment emerged as central determinants shaping implementation sustainability across the participating facilities.

The findings suggest that successful scale-up of neonatal digital health innovations requires governance-oriented and system-sensitive implementation strategies that extend beyond pilot deployment. Leadership engagement, contextual adaptation, workflow integration, and phased hybrid implementation approaches may represent particularly important enabling factors within resource-constrained neonatal settings.

Overall, the study highlights the importance of aligning technological innovation with health system readiness, organizational capacity, and implementation governance to support sustainable institutional integration of neonatal digital health tools in LMIC contexts.

## Data Availability

The raw data supporting the conclusions of this article will be made available by the authors, without undue reservation.
